# New perspectives in allergen specific immunotherapy driven by big trials with house dust mite sublingual SQ^®^ tablets

**DOI:** 10.1186/s12948-020-00124-7

**Published:** 2020-06-11

**Authors:** Gianfranco Vitiello, Lucia Maltagliati, Oliviero Rossi

**Affiliations:** 1 Experimental and Clinical Medicine Department, University of Firenze, Largo Brambilla 3, 50100 Florence, Italy; 2 SOD Immunoallergologia, Azienda Ospedaliero-Universitaria Careggi, Florence, Italy

**Keywords:** Allergen immunotherapy (AIT), House-dust mite, Fast-dissolving tablet, Allergic asthma, Big trials, Asthma guidelines

## Abstract

House-dust mites (HDM) allergy is the prevailing condition in subjects allergic to inhalants. Clinical studies with HDM extracts—either subcutaneous (SCIT) or sublingual (SLIT) have long been characterized by small sample size, varying allergen doses, and poorly defined endpoints assessing disease severity. In the last decade, well-designed, randomized, controlled studies recruiting thousands of patients have been conducted with newly developed HDM sublingual tablets (SQ^®^-HDM tablets). This drug is easily dispersible in the oral cavity due to the patented Zydis^®^ technology and its allergen composition is balanced in terms of group I and group II major mite allergen content, reflecting the equal contribution of the two components to HDM sensitization. HDM is the most common allergen associated with asthma. Clinical efficacy of the SQ^®^ HDM SLIT-tablet in HDM allergic asthma has been evaluated in randomized, double-blind, placebo-controlled trials. Both endpoints related to “present” asthma control (inhaled corticosteroid—ICS) as well as endpoints related to “future” asthma control (occurrence of asthma exacerbations) were included in these studies, in agreement with GINA (Global Initiative for Asthma) guidelines. Based on the positive results of these studies, SQ^®^-HDM SLIT-tablets were approved Europe-wide as registered drug for treating moderate-to-severe allergic rhinitis with or without allergic asthma and not well controlled HDM allergic asthma, associated with allergic rhinitis of any severity. GINA guidelines in 2017 included SLIT-tablet-based immunotherapy as an “add-on” treatment for asthmatic patients sensitized to HDM; indeed, allergen immunotherapy (AIT) is considered to be a complementary treatment option that targets the immunological of allergic diseases, representing the only treatment potentially disease-modifier or, at least, with a long-term efficacy. The availability of a safe, standardized, registered treatment for HDM respiratory allergies is pivotal in the immunotherapy field, pushing it out of a century-long limbo of amatorial interest towards the full dignity deserved by the only casual treatment of respiratory allergies.

## Background

House dust mite (HDM) is the most important respiratory allergen worldwide [1] and *Dermatophagoides* species has long been recognized as the producer of house dust allergens [2].

In agreement with the notion that rhinitis and asthma are expression of the same pathologic condition, continuously affecting the upper and lower respiratory tract (“one airway, one disease”) [3], the presence of isolated allergic rhinitis is considered a risk factor for the subsequent development of asthma [4]. Notably, the relative risk of developing asthma for subjects with allergic rhinitis is higher in case of allergy to mite as compared to other inhalant allergens [5].

**Fig. 1:**
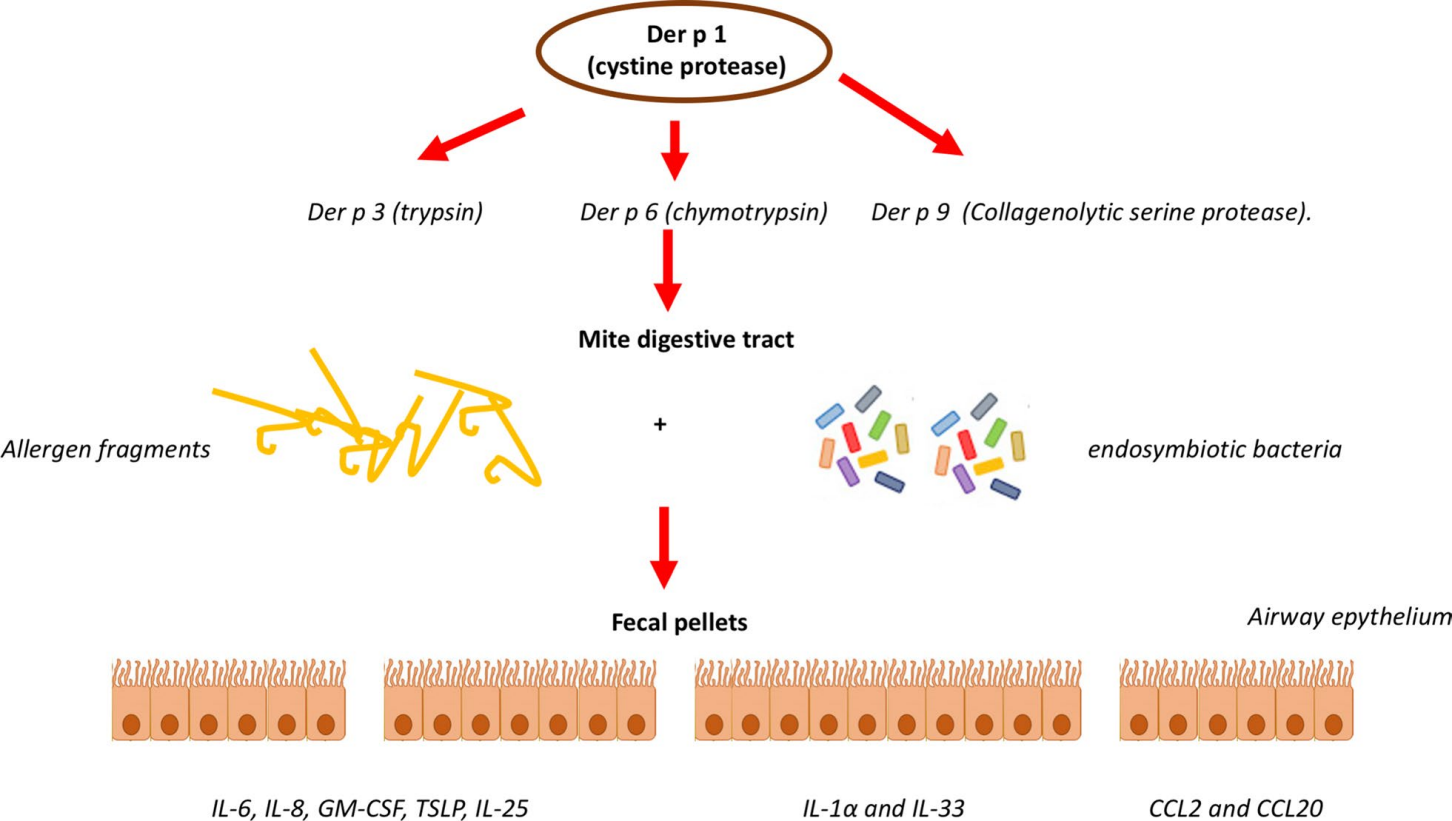
Der p 1 (cystine protease) is a proteolytic trigger of an activation cascade involving several other allergenic proteins, including Der p 3 (trpypsin), Der p 6 (chymotrpypsin), and Der p 9 (Collagenolytic serine protease). These enzymatic activities facilitate the release of mite allergens into the digestive tract of the mite and affects the generation of protein fragments available to antigen processing. Microbial compounds from endosymbiotic bacteria also participate to the formation of fecel pellets, which are expelled onto the airway epithelium. Release of proinflammatory cytokines (IL-6, IL-8, GM-CSF, thymic stromal lymphopoietin, and IL-25), alarmins (IL-1α and IL-33) and chemoattractants (CCL2 and CCL20) by airway epithelial cells follows

The pathogenetic mechanism underlying the allergenicity of dust mite allergens has been the object of basic immunology investigations, which shaped some of the pillars of our present knowledge of the immune system [6]. Indeed, IL-3, IL-4, IL-5 and granulocyte macrophage-colony stimulating factor (GM-CSF) were identified in the supernatants of *Dermatophagoides*-stimulated CD4^+^ T lymphocyte clones in patients with severe atopic disorders. Moreover, allergens such as Der p 1 and Der p 9 play a crucial role at the cross-road between innate and adaptive immunity, since the enzymatic activity of these proteins affect dendritic cells-epithelial cells interactions, induce the cleavage of tight-junction molecules [7] (Fig. 1 ) and activate an inflammatory cascade involving reactive oxygen species (ROS) production and NF-KB activation, ultimately promoting the differentiation of Th2 and Th17 lymphocytes [8]. Recently 44 and 53 antigens have been recognized by cross-immune electrophoresis in extracts from *D. farinae* and *D. pteronyssinus*, respectively [9].

In addition, the link between innate and adaptive immunity is specifically involved in asthma pathogenesis: enzymatically active allergens, sampled on the respiratory epithelia, have been demonstrated capable to gain access to dendritic cells either directly or through cleavage of tight junctions, with engagement of Toll-like receptors and induction of beta-defensins in the context of a cytokine storm involving trypsin-like serine protease (TSLP), GM-CSF, IL-6, IL-25, IL-33 and osteopontin [10], secreted by a complex set of cellular types collectively known as group 2 innate lymphoid cells (ILC2) where a specific role in barrier immunity is played by TSLP secreted by epithelial cells of skin, gut and lung challenged by feces-derived allergen component Der p 1 [7].

Notably, besides allergens, other epithelial danger signals, the so-called “alarmins”, were indicated as cofactors of the inflammatory cascade resulting in Th2 differentiation driven by IL-4, IL-5 and IL-13 and enhanced eosinophils survival, activation and migration to airways. Bronchial hyper-responsiveness and increased mucus production are the functional respiratory correlates of these cellular and molecular events [11].

## Mechanisms of action of immunotherapy

Mechanisms underlying AIT are complex and only partially understood. Allergen-specific T cell help is required for priming the synthesis of allergen-specific IgE antibodies leading clinical symptoms of allergy. Once established, the secondary allergen-specific IgE and T cell response are modulated by allergen-specific IgE and IgG antibodies. Allergen-IgE complexes activate mast cells and basophils and perpetuate allergen-specific T cell responses via IgE-facilitated antigen presentation (FAP) [12–14]. Moreover, specific IgE can directly activate IgE memory B cells [15].

Allergen-specific IgG antibodies induced by AIT likely interfere with these processes by binding to IgE binding sites of allergens, thus counteracting both immediate and delayed allergic inflammation and specific IgE production [15]. Evidence of allergen-specific immune modulation were found in patients treated with SQ^®^-HDM tablets. A clear dose-related increase in HDM specific IgG4 antibodies was found in studies for the active treatment, while no evidence for such an effect was observed in the placebo group [16]. This observation supports the notion that the immune system is modulated by treatment with SQ^®^-HDM, which may explain the pathogenic mechanisms of the observed clinical effects in terms of down-regulation of Th2 immunity.

## Outcomes of immunotherapy with sublingual tablets

Safety and efficacy of AIT has long been investigated. Both sublingual (SLIT) and subcutaneous (SCIT) AIT have received much attentions over the last decades, and symptom reductions and safety of administration have been investigated as clinically relevant end-points. SLIT presents a relatively better safety profile in comparison with SCIT, that has been associated to a higher risk of systemic reactions, including anaphylaxis [17], even though the overall rate of adverse reaction is similar between the two regimen [18]. It has been established that 3 to 5 years of treatment are necessary to achieve and consolidate clinically relevant results, which brings about the issue of low adherence, as it is the case for any chronic treatment [19]. Beside its favorable impact on symptoms deriving from allergen exposure, AIT also prevents the appearance of new sensitizations [20].

A comprehensive revision of literature in 2013, after careful analysis of 44 studies with HDM immunotherapy, highlighted the marked interstudy heterogeneity (end-points, reporting, inclusion criteria, doses, formulations, duration of treatments), identified several potential improvements to be implemented and concluded that more definitive trials were needed [21]. Nevertheless, the strong rational of AIT, as the only causal treatment of allergy, has never been questioned. Mechanisms of immune tolerance relies on the induction of regulatory T cells and increased production of IL-10 and TGF-beta at the sites of allergic inflammation [20, 21] and the possibility to induce a permanent suppression of Th-2 inflammation was considered compatible with available evidence [22].

The first signs that revealed the appearance of a new era in AIT can be dated back to the first years of the millennium, when grass tablets were formulated and tested for safety and efficacy with double-blind, placebo-controlled studies in a number of patients comparable to those recruited in the registration process of any drug, i.e., approximately ten times higher than in typical studies with immunotherapy extracts performed until then [23]. Two products, the 5-grass pollen sublingual tablet and the 1-grass pollen sublingual tablet were, therefore, licensed as a drug. The large number of clinical trials published allowed the formal demonstration of the disease modifying effect of AIT, that is the persistence of the positive effect of symptoms and symptomatic drug consumption after interruption of immunotherapy [24]. Indeed, grass allergy can be associated with asthma, but this allergen is not a per se risk factor for developing asthma in non-asthmatic allergic subjects, neither a risk factor for more severe asthma, as it is the case for HDM [25]. However, in 2018 a complex large scale, placebo-controlled study in children with rhinitis and no asthma demonstrated that 3-years AIT with grass registered tablet reduced the appearance of asthma symptoms and medication use in 2 years follow up time, including out-of-season months. With this trial the impact of AIT on the natural history of allergy as a systemic disease, which was previously reported in small-scale, open studies [26], could be confirmed within an experimental setting fully adherent with the international guidelines for good clinical practice [27].

Overall, allergen immunotherapy exemplifies the intervention perfectly fitting to patients with defined immunological profiles and represents a model of “precision medicine”. Indeed, on the one side etiologic agents, pathophysiology and symptoms of allergy have been dissected at molecular level, and on the other immunotherapy has demonstrated the potential to interfere specifically on the natural history of the disease [28].

A few years after the registration of the first grass tablet for immunotherapy, the strategy for the development of the Standardized Quality (SQ^®^) HDM (HDM) sublingual immunotherapy tablet was initiated. The distinct characteristics of the SQ^®^—HDM tablet are summarized in Table 1.

**Table 1:** Characteristics of the SQ^®^-HDM registered tablet [9]

Proteins from *D. pteronissynus*, percent	50%
Proteins from *D. farinae*, percent	50%
Proportion of group I/group II allergens	1/1
Der p 1 + Der p 2 content	15 µg
Der f 1 + Der f 2: content	15 µg
Formulation (orodispersible)	Zydis^®^ technology
Time to full dissolution in oral cavity	3–4 s

After concluding phase I studies, SQ^®^-HDM tablet were investigated in clinical trials evaluating efficacy and safety both in allergic rhinitis and in allergic asthma.

## HDM Allergic asthma

In the MT-02 study Mosbech and co-authors [29] recruited 604 patients with allergic asthma and allergy to mites, and randomly assigned them to one of 4 different arms, i.e., placebo, 1, 3 or 6 SQ^®^-HDM units. Before taking of placebo or investigational drug, patients were assessed by the Asthma Control Questionnaire (ACQ) with the aim to obtain a satisfactory level of asthma control with budesonide Turbuhaler™. This formed the basis of the baseline measurement for the primary end point. The ICS adjustment process was repeated, with the end-of-trial ICS stable period—placed during the winter—forming the basis for the primary efficacy assessment. After 1 year of treatment, patients who had received 6 SQ^®^-HDM required a significantly lower amount of ICS (average 81 µg per day, corresponding to a 42% reduction versus controls) in order to achieve the target level of asthma control (ACQ < 1,5).

The ICS reduction was also analyzed in the subgroup of patients with not well-controlled asthma on medium–high doses of ICS [30], i.e., daily ICS use of 400–800 µg budesonide and ACQ score 1–1.5. This analysis included 108 subjects evenly distributed in the 4 treatment arms and demonstrated a substantially higher and statistically significant effect in the subgroup that had received 6 SQ^®^. By the selection, the average mean use of ICS in the baseline period was higher in this subgroup. In the placebo group, the ICS use was reduced by 9% versus baseline in the efficacy assessment period, whereas in the 6 SQ^®^-HDM group reduction of ICS use reached 63%. This corresponded to a highly statistically significant difference between placebo and 6 SQ^®^-HDM in daily ICS use (327 µg). This analysis demonstrated that the effect of AIT was more clinically relevant in the subgroup of patients with uncontrolled disease. In a post hoc analysis of such study [30], the total score of the rhinitis quality of life questionnaire with standardized activities RQLQ(S) was used as a secondary end-point. This parameter, clinically reflecting the impact of treatment on everyday patients’ life, was evaluated for 5 individual domains: activities, sleep, non-nose and non-eye symptoms, nasal symptoms. A significant reduction was observed in the 6 SQ^®^-HDM group, both in terms of overall quality of life and within single domains. Notably, both total combined rhinitis score (TCRS) and quality of life parameters displayed a dose response pattern, with lower, although nonsignificant differences for 1 and 3 SQ^®^-HDM, further supporting—beside placebo comparison—the notion that the observed impact on rhinitis depended on the immunological effect of the investigational drug. No safety concerns were observed.

**Fig. 2:**
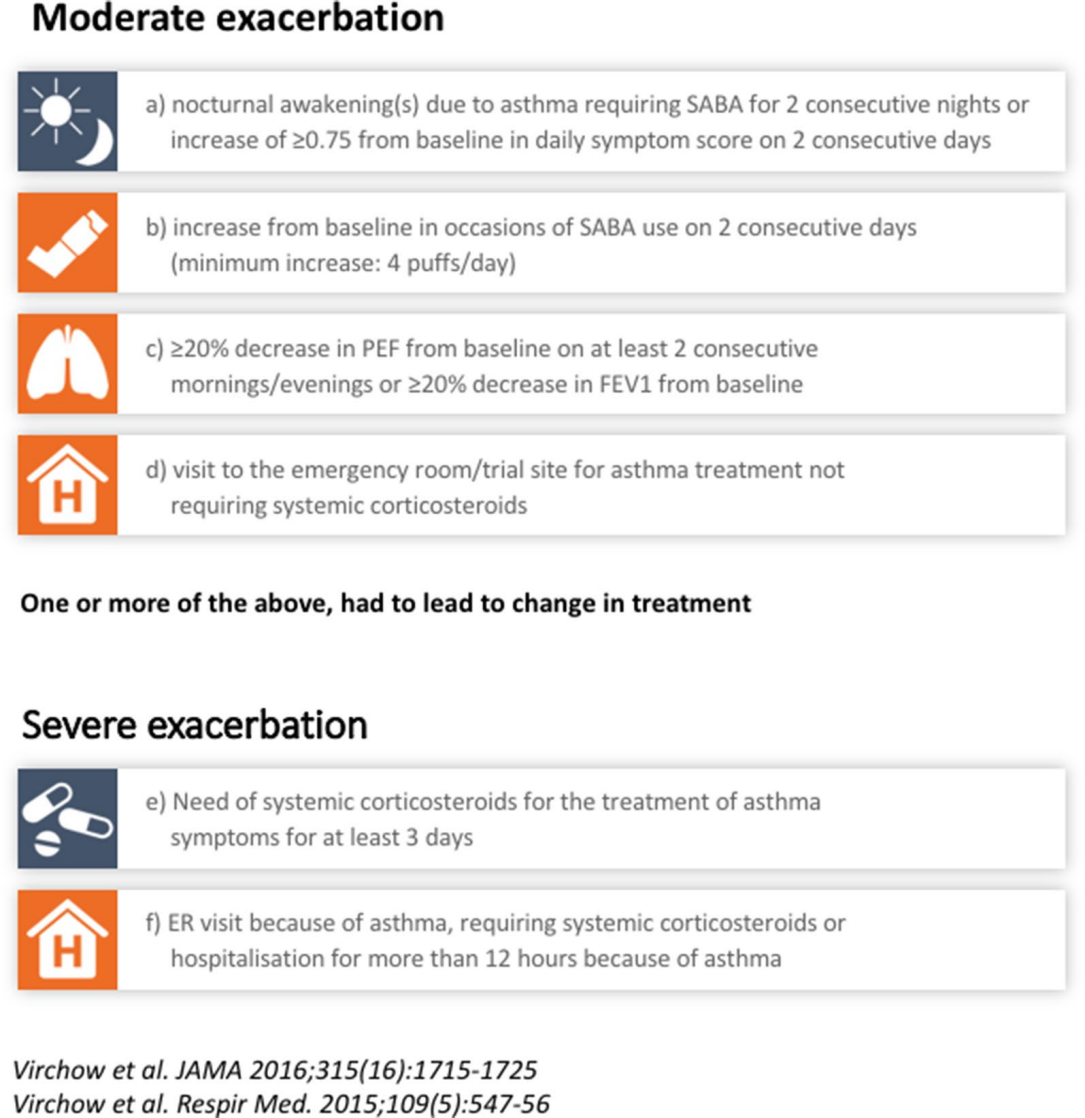
Definition of asthma exacerbation in the MITRA trial

Having MT-02 met the primary outcome in terms of reduction of ICS in the asthmatic sub-population with only partial symptom control, the design of a subsequent phase III study, the MITRA trial [16] was planned accordingly. In this study, eligible patients were adults with a positive result for HDM specific serum IgE and/or skin prick test and a clinical history of more than 1 year of allergic asthma and allergic rhinitis. In recruited patients, HDM was considered clinically as a major trigger and asthma was not well controlled by ICS (equivalent to budesonide, 400–1200 μg) at inclusion. The forced expiratory volume in the first second of expiration (FEV1) at randomization was 70% or more of predicted value. The ACQ score required for entering the study was 1 to 1.5 (score range, 0–6; values below 1 = controlled asthma, values above 1.5 = uncontrolled asthma). Hospitalization due to an asthma exacerbation within 3 months prior to randomization was an exclusion criterion. Participants could have multiple sensitizations but were not allowed to have a relevant clinical history of perennial allergic asthma or rhinitis caused by other allergens. The MITRA trial was a randomized, placebo-controlled, parallel-group, double-blind trial evaluating the efficacy and safety of 1-year treatment with 6 and 12 SQ^®^ HDM-tablets. The trial was performed in 109 sites in 13 European countries. The trial Asthma controller medication, when taken by patients at study entry, was switched to budesonide (400–1200 mcg) and salbutamol on demand. 834 patients were randomized (1:1:1) to receive placebo, 6 SQ^®^-HDM tablets, or 12 SQ^®^-HDM tablets for up to 18 months, as add-on therapy to ICS and salbutamol. After 1 year of treatment, ICS treatment was reduced 50% for 3 months, followed by a complete withdrawal for 3 more months in patients who did not experience asthma exacerbation. Primary outcome was time to first moderate or severe asthma exacerbation during the ICS reduction period. An asthma exacerbation was defined according to the American Thoracic Society and European Respiratory Society (ATS/ERS) recommendation; and described by 1 or more of the criteria for moderate or severe asthma exacerbation leading to a change in treatment. Definition of moderate and severe asthma exacerbations [16, 31] are shown in Fig. 2.

This Kaplan–Meier plot of the primary efficacy analysis showed a statistically significant reduced risk for asthma exacerbations for both treatment doses versus placebo. The pre-defined clinically relevant effect size was a hazard ratio equal to or below 0.7 (corresponding to a risk reduction of 30% or more). This was met for both 6 and 12 SQ^®^-HDM doses for the full analysis set. More and earlier occurring asthma exacerbations were observed in the placebo group, with separation of the effect between placebo and active being apparent immediately after ICS reduction. The actual time to the first exacerbation experienced by 25% of the subjects was around 100 days for placebo, 170 days for 6 SQ^®^-HDM and above 180 days for 12 SQ^®^-HDM. HDM SQ^®^ tablets also reduced the risk of a first moderate to severe asthma exacerbation associated with deterioration on asthma symptoms or nocturnal awakening, which was the secondary key endpoint.

Taken together, MT-04 and MITRA trials demonstrated that AIT is highly effective in reducing ICS use, future exacerbation rates and overall asthma symptoms, with a good safety profile. It is important to notice that asthma guidelines consider these very features as pivotal for the choice of the best treatment for the disease.

## HDM allergic rhinitis

Efficacy of SQ^®^-HDM tablets in treating the symptoms of mite-induced allergic rhinitis was investigated in 12 European countries with the MERIT trial [32]. Adults aged 18 to 65 years with a history of moderate to severe HDM-induced rhinitis, with or without conjunctivitis and asthma were randomized to receive the investigational drug (N = 318) or placebo (N = 338) for 1 year. One of recruitment criteria required that subjects had a total daily rhinitis symptom score of a least 6 and had to use symptomatic medication during at least 8 days of the 15 days baseline period. Also, at least one of the ARIA quality of life items—sleep disturbance, impairment of daily activities, leisure and/or sport or impairment of school or work—had to be present during the baseline period. Thus, this trial population represented subjects with significant allergic disease who were substantially bothered by their symptoms and not adequately controlled by symptomatic medications. During treatment period of approximately 10 months the subjects received the SQ^®^ HDM SLIT-tablet or placebo for daily administration and were provided with nasal steroid, oral antihistamine, and antihistamine eye drops to be used as needed.

During the efficacy assessment period the subjects completed the diary (rhinitis symptoms and medication use), which allowed to measure the primary endpoint: TCRS averaged over the last 8 weeks of treatment.

The rhinitis symptom score consisted of 4 symptoms; runny nose, blocked nose, sneezing and itchy nose assessed on a scale from 0 to 3 with 0 being no symptoms and 3 being severe symptoms. The maximum daily rhinitis symptom score was thus 12. The rhinitis medication score was calculated based on the use of Desloratadine tablets and/or Budesonide nasal spray. The maximum daily use of Desloratadine tablets was 1 tablet which was assigned a score of 4. The maximum daily use of budesonide nasal spray was 1 spray per nostril twice daily giving a maximum daily score of 8. Thus, the maximum daily rhinitis medication score was 12. So, the maximum daily TCRS was 24, with a 1:1 balance between symptom and medication scores.

In the phase III trial MT-06 [32], both 6SQ^®^-HDM and 12 SQ^®^-HDM doses met the pre-defined criteria of an absolute reduction in TCRS of > 1, with a difference of 1.18 for the 6-unit dose and 1.22 for the 12 SQ^®^-HDM dose. This reduction was statistically significant for both doses. Analysis of TCRS over the entire time course of the trial indicated that the score decreased (i.e., symptoms improved) during the trial in all 3 treatment groups. The score was consistently lower in the two active groups and demonstrated an early onset of action already with significant difference from placebo at week 14.

A large effect seen was observed in the placebo group. This was expected since patients had access to symptom relieving treatments and received guidance on how to use the medication optimally. Therefore, the effect in the active treated groups is likely an add-on effect to optimal symptom relieving treatment. Also, the patients were seen every 8th week, asked about symptoms, adherence and completed diaries regularly. As the patients were a highly selected group according to both symptoms and medication at randomization, regression to the mean (i.e., the possibility that by chance some of them could get better) was also likely to play a role. Finally, the Hawthorne effect (i.e. the real “placebo effect”, the perception/psychological effect of being treated) could have played a role.

Post hoc subgroup analyses of the primary end point showed that there was no statistically significant difference between the treatment effect in patients with asthma versus no asthma and mono-sensitized versus polysensitized subjects [32].

All individual symptom scores (blocked nose, itchy nose, runny nose, sneezing) were statistically significant for the 12 SQ^®^-HDM dose and supported the decision to move forward with this dose.

**Fig. 3:**
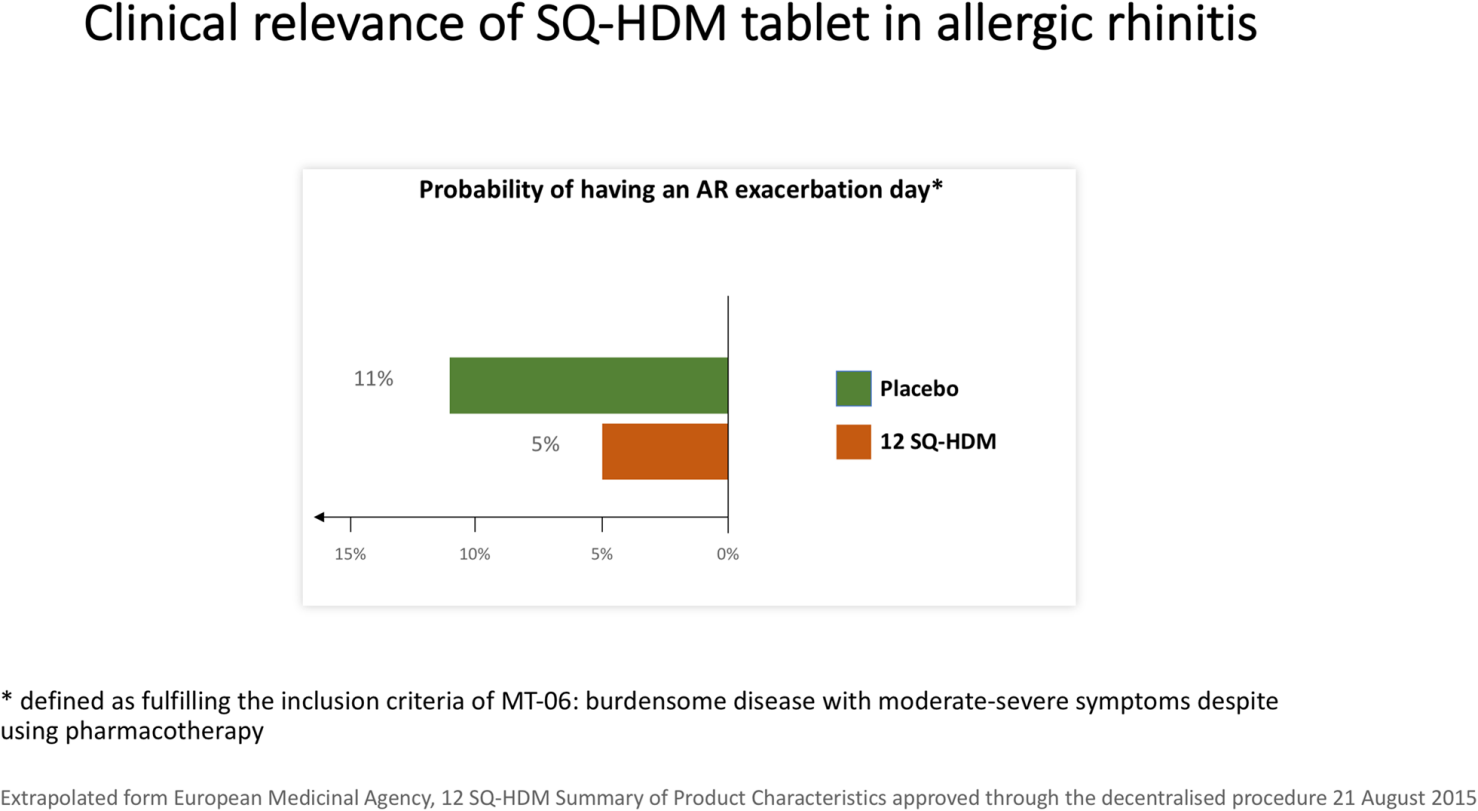
Clinical relevance of SQ-HDM tablet in allergic rhinitis

Clinical relevance of MERIT results was analyzed by considering annual burden of symptoms and medication use. The probability for having an AR exacerbation day, defined as fulfilling the inclusion criteria of MT-06 (burdensome disease with moderate-severe symptoms despite using pharmacotherapy) occurred in 5% in the 12 SQ^®^-HDM dose group vs. 11% in the placebo group. Translating the probability into the actual benefits a patient could get during a full year, the number of days patients report really burdensome symptoms could be halved, namely from average 40 days/year to average 19 days/year (Fig. 3).

A consistent and dose related trend with reduction in all RQLQ domains compared to placebo was also observed in the MERIT trial. In particular, statistically significant effects were found for sleep (primary driver of effect), nasal- and non-nose/eye-symptoms and for impact on every day activities [32], which further highlights the clinical relevance of these results.

**Fig. 4:**
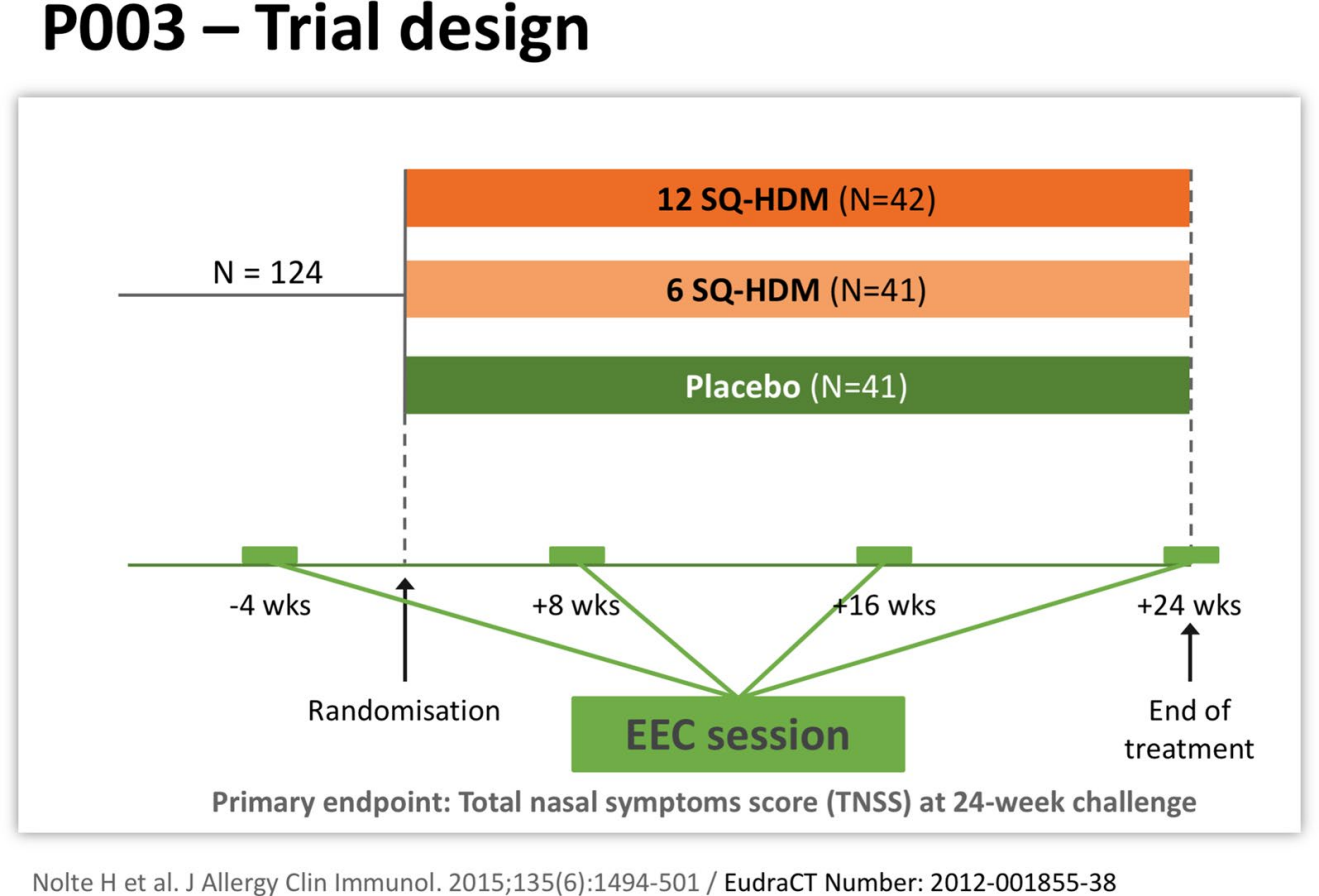
Trial design of the P003 controlled study that evaluated safety and efficacy of SQ^®^-HDM

Another controlled study which evaluated safety and efficacy of SQ^®^-HDM tablet was run in North America (study code: P003) with adult patients, aged 18 to 65, suffering moderate to severe persistent HDM allergic rhinitis of ≥ 1 year’s duration, with or without conjunctivitis, with or without asthma. House dust mite allergy had to be proven by positive skin prick test and/or specific IgE (*D. pteronyssinus* and/or *D. farinae*). A total nasal symptom score of ≥ 6 (out of 12), within the first 2 h of the screening EEC session prior to randomization was an entry criterion [33]. Exclusion criteria were unstable, uncontrolled/partially controlled, or severe asthma as judged by the investigator; asthma requiring medium- or high-dose inhaled corticosteroids within the last 12 months before screening. The P003 trial was performed in the Vienna exposure chamber It was a randomized, placebo-controlled, parallel-group, double-blind, dose-finding trial evaluating the safety and efficacy of 6 and 12 SQ^®^-HDM. The trial included 4 visits to the exposure chamber (Environmental Exposure Chamber, EEC)., each lasting 6 h (Fig. 4).

Three days prior to each chamber session, the participants were required to stop the use of antihistamines as well as decongestants. Use of oral, nasal, or ocular corticosteroids was not permitted during the trial. 124 subjects were randomized into 3 groups of equal sizes. Before randomization, the subjects participated in the first 6-h chamber session. Additional chamber sessions took place after 8, 16 and 24 weeks of treatment. During the sessions, subjects scored their rhinitis, conjunctivitis, and asthma symptoms on a 0–3 scale every 15 min. The primary endpoint was the average total nasal symptom score (TNSS) during the chamber challenge at week 24.

TNSS during the chamber challenge at week 24. Symptoms were quantified as follows: runny nose (0–3), blocked nose (0–3), sneezing (0–3), itchy nose (0–3). Thus, the maximum symptom score was 12. At the end of the trial, the reduction in symptom score was 27% and 49% for the 6 and 12 unit, respectively. A clear dose response was observed for both onset and magnitude of the effect, further supporting the cause-effect relationship of results.

A similar pattern was observed when including also eye symptoms in the analysis. This can be seen as an optimal effect that can be achieved in a controlled setting where allergen exposure is standardized, and no symptomatic medication effects are involved.

The treatment was overall well tolerated, as most of the adverse drug reactions were mild in severity and characterized by oral pruritus and throat irritation. This data clearly supports the superiority of the 12 SQ^®^-HDM dose, which finally entered the market. Taking everything in consideration, HDM SLIT tablets are considered to be a safe therapeutic option in moderate to severe HDM-induced allergic rhinitis [34].

## Adolescents

A DBPC RCT was concluded in North America [35], which assessed the efficacy/safety of HDM SLIT-tablets in patients with HDM-induced allergic rhinitis with or without conjunctivitis (AR/C). 1482 subjects (aged ≥ 12 years) with HDM-induced AR/C with or without asthma were recruited and randomized to a daily SQ^®^ HDM SLIT-tablet (12 SQ^®^-HDM dose) or placebo for up to approximately 52 weeks. A rhinitis daily symptom score of 6 or greater on 5 of 7 consecutive days before randomization was required. The primary end point was the average total combined rhinitis score during the last 8 treatment weeks. Treatment with 12 SQ^®^-HDM improved the total combined rhinitis score by 17% (95% CI, 10% to 25%) versus placebo.

A similar trial, with a lower SQ^®^-HDM dose (6 SQ^®^-HDM) was completed in Japan [36]. 458 Japanese children with symptomatic AR (following symptomatic drug suspension during run in) were randomly assigned to a daily 6 SQ^®^ HDM SLIT-tablet or placebo treatment for 1 year. Both pediatric subjects, aged 5–11 years and adolescents, aged 12–17 years were included. The primary endpoint was the TCRS comprising AR symptom and medication scores during the last 8 weeks of the treatment period. A significant reduction in TCRS of 1.22 with a relative difference of 23% (95% confidence interval, 14 to 31%) was observed in treated subjects versus controls. The same degree of efficacy was observed in children and adolescents. The treatment was also well tolerated by all subjects. Following the positive results of these trials, 12 SQ^®^-HDM tablets indication in Europe was extended to rhinitis also for adolescents aged 12-17.

## Adverse events observed during clinical studies with SQ^®^-HDQ tablets

Overall, in studies with SQ^®^-HD tablets the percentage of subjects with treatment related adverse events was clearly dose dependent [16, 32]. In the MT-06 trial, more subjects in the 2 active groups reported study drug-related adverse events compared to placebo. In most cases, symptoms were mild to moderate with no interference to moderate interference with subjects’ daily activities. The most frequently reported treatment-related adverse events were local reactions in mouth and throat such as oral pruritus, throat irritation, and edema of the mouth, which all had a fast onset within the first 1–2 min after first tablet intake. The resolution of the most frequent events was within a few minutes similarly to most sublingual immunotherapy treatment for respiratory allergies. Also observed rates of discontinuation (3–6%) due to treatment related adverse events were similar to what observed in development programs with immunotherapy products.

Adrenaline was administered to one patient presenting mild laryngeal oedema within 5 min after first tablet intake. The subject experienced palatine and oropharyngeal pruritus followed by dysphonia, throat irritation and dry cough. For this reason, he was treated with adrenaline (1‰, 0.5 ml intramuscular), 32 mg oral methylprednisolone and 5 mg oral desloratadine. Physical examination did not reveal any abnormalities and all symptoms abated after 30 min. The subject, however, was able to complete the trial without other AEs, except for mild oral pruritus [32].

## GINA guidelines

In 2017 for the first time GINA guidelines highlighted the need to treat the allergic component of asthma. Sublingual immunotherapy was recommended as add-on treatment for patients with HDM allergic asthma (steps 3 and 4) (Global Initiative for Asthma-GINA-2018 GINA Report, Global Strategy for Asthma Management and Prevention) [37]. Recommendation was based on the results of the MITRA trial with 12 SQ^®^-HDM tablets that demonstrated improved asthma control [16] and was expressed as follows: “Consider adding SLIT in adult HDM-sensitive patients with allergic rhinitis who have exacerbations despite ICS treatment, provided FEV1 is > 70% predicted”.

AIT makes its official entry into the field of asthma management by preserving symptom control while minimizing the side effects of classic treatment. Indeed, the Guidelines highlight the possibility of a step-down of the therapy after a prolonged exacerbation-free period. In this context, AIT con be considered to facilitate step-down in patients with HDM sensitization in therapy with medium dose inhaled corticosteroids [38].

## Conclusions

The SQ^®^ HDM SLIT-tablet has proven an efficacious etiological treatment in HDM AR and AA, also when both manifestations are present simultaneously. Primary endpoints met in both the AR and AA clinical studies can be translated to significant clinical benefits for patients with moderate to severe disease who are not controlled by pharmacotherapy. Efficacy was observed after 8–14 weeks of AIT, and its effect was sustained throughout the year of treatment.

Treatment was well-tolerated and compatible with home administration, with the exception of the first dose, that must be taken under physician supervision.

SQ^®^-HDM tablets treatment of respiratory allergy to mites is associated with measurable changes in the immune response to allergens, reduction of the risk of exacerbations, improvement of disease control, reduction of symptomatic pharmacotherapy, favorable impact on the quality of life of patients [16, 32, 38]. Last but not least, immunotherapy has the potential to provide a long-term clinical benefit even after cessation of treatment [39].

## Data Availability

Not applicable.

## Abbreviations

AA: Allergic asthma
AR: Allergic rhinitis
ARIA: Allergic rhinitis and its impact on asthma guidelines
GINA: Global initiative for asthma guidelines
HDM: House dust mite
ICS: Inhaled cortico steroids
ILC2: Group 2 innate lymphoid cells
RQLQ: Rhinitis quality of life questionnaire
SmPC: Summary of product characteristics
SQ^®^: Standardized quality
TNSS: Total nasal symptom score
